# Characteristics of soil origin Pseudomonas batumici Koz11 isolated from a remote island in Japan

**DOI:** 10.1099/acmi.0.000799.v3

**Published:** 2024-08-16

**Authors:** Hui Zuo, Yuh Morimoto, Kenzo Muroi, Tadashi Baba

**Affiliations:** 1 Antimicrobial Resistance Research Center, National Institute of Infectious Diseases, 4-2-1 Aoba-cho, Higashimurayama-shi, Tokyo, Japan; 2 Department of Radiological Technology, Faculty of Health Science, Juntendo University, 2-1-1 Hongo, Bunkyo-ku, Tokyo, Japan; 3 Graduate School of Nursing, Seisen Jogakuin College, 2277 Kurita, Nagano, Japan

**Keywords:** antimicrobial activity, *P. batumici*, soil microorganisms

## Abstract

Soil samples from a remote Japanese island (Kozushima) were processed and investigated for organisms exhibiting antimicrobial activity against pathogenic strains. A *Pseudomonas* strain demonstrating antimicrobial activity against *Staphylococcus aureus* (*S. aureus*) was identified, prompting further investigation. Whole-genome sequencing was employed to identify the species and conduct phylogenetic analysis, followed by *in silico* molecular analysis. Chemotaxonomic and biochemical analyses were conducted to further characterize the strain. Genomic analysis identified the strain of interest as *Pseudomonas batumici* (*P. batumici*), originally isolated from soil of the Black Sea coast of the Caucasus in 1980. *P. batumici* Koz11 is the second *P. batumici* strain to be isolated and identified outside its initial area of discovery. Similar to the type strain, *P. batumici* Koz11 showed antimicrobial activity against various *S. aureus* strains, including methicillin-resistant *S. aureus* (MRSA) and vancomycin-resistant *S. aureus* (VRSA). However, the previously reported ‘batumin gene cluster’, which synthesizes antimicrobial compounds, was absent from *P. batumici* Koz11. This study provides new insights into *P. batumici*. Since the type strain of *P. batumici* is exclusively deposited in the Ukrainian Collection of Microorganisms, the Koz11 strain may serve as a surrogate to facilitate continued study of *P. batumici*.

**Significance as a BioResource to the community**

Our investigation focused on *P. batumici* strain Koz11, the second isolate of its kind, identified from soil samples. Utilizing long-read sequencing, we obtained a complete circular genome for this strain. Additionally, we conducted chemotaxonomic and biochemical analyses, as well as *in silico* investigations, which had not been fully characterized previously. Despite the absence of the ‘batumin gene cluster’ observed in *P. batumici* UCM B-32^T^, the Koz11 strain demonstrated notable antimicrobial activity against *S. aureus* strains, including MRSA and VRSA. This underscores the potential significance of *P. batumici* in synthesizing several antimicrobial substances.

## Data Summary

All supporting data and accession numbers are provided within the article or through supplementary data files. The sequence data of strain Koz11 was deposited in GenBank databases under the GenBank accession number CP144470 and BioProject accession number PRJNA867113. The Koz11 strain has been deposited in the Spanish Type Culture Collection under the accession number CECT 9964.

## Introduction

Soil ecosystems host a wide range of micro-organisms. While soil can act as a reservoir for diseases, it also harbours a diverse array of microbes that play a crucial role in sustaining human, animal and plant life [1,2]. In recent decades, the concept of soil health has evolved, bringing increased attention to the ability of biological organisms to suppress pathogens, in addition to facilitating crop production [1,3]. The coexistence of pathogenic microbes and antimicrobial substances produced by soil micro-organisms suggests that the soil ecosystem is dynamically regulated, potentially impacting human and animal health.

This study emerged from an exploration of the impact of soil-origin microbes on human and animal pathogens. Among the multitude of soil microbes, we focused on *Pseudomonas* spp. due to their ubiquitous presence in natural environments including soil and their well-known capability to produce various secondary metabolites with a broad range of biological activities [4,6]. Soil environments exhibit concentration gradients of essential elements for growth, leading to the intense competition between organisms that share the habitat [6]. Consequently, some secondary metabolites possess antimicrobial activities that suppress competitors, including pathogenic organisms [7,9].

The present study describes a *Pseudomonas* spp. strain found in a soil sample collected from Kozushima, a remote island in Tokyo (N34° 205′, E139° 134′). The organism exhibits antibacterial activity against *Staphylococcus aureus*, one of the major human and animal pathogens [10]. Whole-genome sequencing revealed that the *Pseudomonas* spp. strain from Kozushima belongs to the species *Pseudomonas batumici*, which was first isolated from soil along the Black Sea coast of the Caucasus. * P. batumici* UCM B-321^T^ is known to produce an antibiotic compound called ‘batumin’ (also known as ‘kalimantacin’), which has been extensively studied by a Ukrainian research group [11,14]. The Kozushima-origin *P. batumici* Koz11 is the second strain of * P. batumici* isolated and identified beyond the region where it was first found in 1980. This strain exhibits similar antimicrobial activities; however, the biosynthetic gene cluster responsible for producing batumin was not observed. This article provides insights into the chemotaxonomic and biochemical traits of *P. batumici* that have not been fully characterized to date, facilitating better comparisons with closely related species. The article also presents a simple isolation method for soil-origin *Pseudomonas* strains, as well as a rapid antimicrobial activity test method that requires only basic microbiology laboratory equipment and free software. Given that the type strain of *P. batumici* is exclusively deposited in the Ukrainian Collection of Microorganisms, Koz11 may serve as a surrogate to advance research on this organism and its potential benefits.

## Methods

### Bacterial isolation from soil samples

**Fig. 1.**
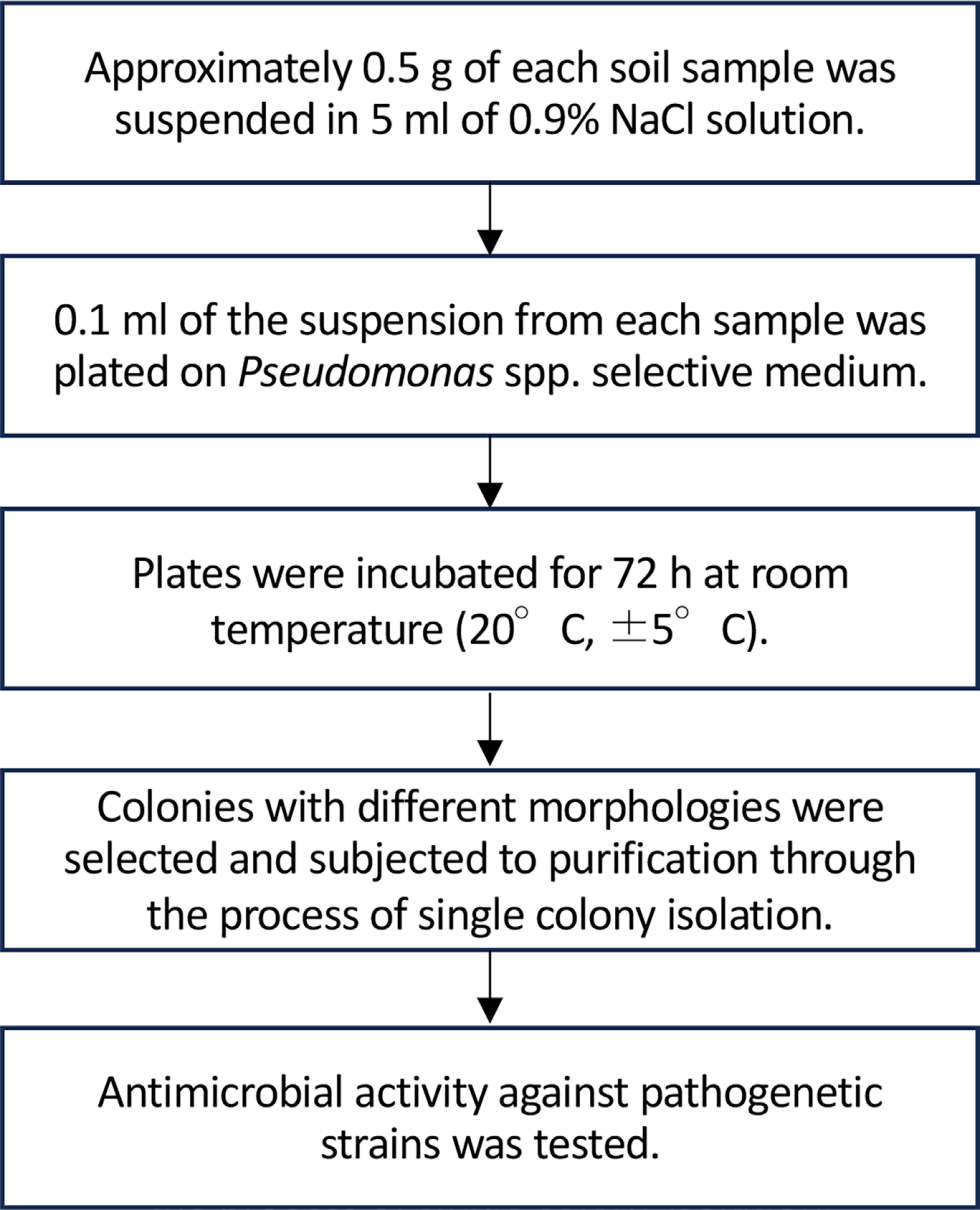
Procedure for the isolation of *P. batumici* Koz11 from soil samples.

Bacterial isolates were obtained from soil samples collected at six locations on Kozushima Island in July 2017. The locations were the west-coast beach, southeast passage, the southeast observatory, the seventh climbing stage of Mt. Tenjo, the north-coast beach and the edge of the pond at the summit of Mt. Tenjo. Approximately 0.5 g of each soil sample was suspended in 5 ml of 0.9 % NaCl solution. Next, 0.1 ml of the suspension from each sample was individually plated on *Pseudomonas* spp. selective medium (*Pseudomonas* CFC/CN agar, Merck). The plates were incubated for 72 h at room temperature. Subsequently, colonies with different morphologies were selected and subjected to purification through the process of single colony isolation (Fig. 1 and Fig. S1a, available in the online version of this article).

### Pathogenic bacterial strains

Bacterial strains used in this study included *S. aureus* strains FDA209P, USA300FPR3757, N315, MW2, COL, VRS1, Mu50 and NCTC8325, as well as *Escherichia coli* (*E. coli*) strain 1708, as previously described [15].

### Antimicrobial activity test

To assess the antimicrobial activity of the Kozushima strain, a few colonies of a pathogenic bacterial strain were selected from nutrient agar medium (Eiken Chemical Co., Ltd., Tokyo, Japan) and suspended in saline. After adjusting the suspension to a McFarland standard of 0.5, the strain was spread on fresh nutrient agar medium using a swab. Colonies from the Kozushima isolate were transferred using a sterilized toothpick and inoculated onto the nutrient agar plate where the pathogenic strain had been spread. The plate was incubated overnight at room temperature, followed by an additional 4 to 6 h at 37 °C to promote the growth of the underlying pathogenic strain (the procedure is illustrated in Supplementary Fig. S1b). The experiments were repeated three times to ensure reproducibility. They were also performed on trypticase soy agar (Becton Dickinson, MD, USA) plates and Mueller–Hinton agar (Nippon Becton Dickinson, Co., Ltd., Tokyo, Japan) plates to validate the results across different culture media. The nutrient agar plates with inhibition zones were then photographed using the FAS-IV system (Nippon Genetics Co., Ltd., Tokyo) under light-emitting diode mode, and the images were saved in 8-bit TIFF format. These image files were analysed to measure the size of the inhibition zones using ImageJ software [16]. The pixel-based length from the horizontal edge of the inhibition zone to that of the Koz11 colony was obtained, and the length in millimetres (mm) was calculated using the diameter of the plate (90 mm, 960 pixels). The mean length of eight inhibition zones obtained from the two independent experiments was reported. A step-by-step description of the measuring procedure is given in Supplementary Fig. S1c. Minimum inhibitory concentrations for *S. aureus* strains were determined using the microdilution method with Dryplate Eiken DP32 (Eiken Chemical Co., Ltd., Tokyo, Japan). Since the eight *S. aureus* strains have been extensively studied [17], making the results easily comparable to previous research, minimum inhibitory concentration tests were performed once.

### Bacterial identification

Genomic DNA was extracted using QIAamp DNA Mini Kits (Qiagen, Hilden, Germany) following the manufacturer’s instructions. The DNA sequence of the 16S rRNA gene was analysed with the BigDye Terminator v3.1 Cycle Sequencing Kit and the ABI PRISM 3100 Genetic Analyzer (Applied Biosystems, Life Technologies, Carlsbad, CA), using primers 8F (5′-AGAGTTTGATCCTGGCTCAG-3′) and 1541R (5′-AAGGAGGTGATCCAGCCGCA-3′). For whole-genome sequencing, the sample was prepared using the Pacific Biosciences (PacBio) SMRTbell Express template prep kit, and the genome was sequenced using PacBio RS II SMRT platform at the Macrogen (Tokyo, Japan). Raw read quality was assessed using SMRT Portal (v2.3), and *de novo* assembly was performed using CLC Genomics Workbench v7 (Qiagen) and the Hierarchical Genome Assembly Process three pipeline.

### Species determination

The 16S rRNA gene sequence was compared with type strains using the EzTaxon-e database [18]. Whole-genome sequence was compared with reference sequences using average nucleotide identity (ANI) using the ANI calculator provided by EzBioCloud [19], and digital DNA–DNA hybridization (dDDH) was performed using the Genome-to-Genome Distance Calculator [20].

### Chemotaxonomic and biochemical characterization

Fatty acid methyl ester analysis was performed at Techno Suruga Laboratory Co., Ltd. (Shizuoka, Japan). The strains were cultured on tryptic soy agar plates at 28 °C for 24 h. Fatty acids were prepared and analysed using microbial identification (Sherlock MIDI 6.0) [21]. The biochemical characteristics of Koz11 were examined using Biolog GEN III MicroPlates and API 20 NE strips (bioMérieux), following the manufacturer’s instructions. These experiments were performed once. To ensure valid results, the procedures recommended by the manufacturer’s instructions were strictly followed, including using pure cultures of Koz11 that had undergone single colony isolation and using disposable sterile products to avoid any trace amounts of detergent [22].

### Additional molecular phylogenetic analysis

To determine the phylogenetic relationship between the Koz11 strain and 33 representative type strains, phylogenetic trees were generated using Bacterial Genome Tree service provided by BV-BRC (https://www.bv-brc.org/) [23,24]. In short, amino acid and gene nucleotide sequences from the chosen genes in BV-BRC’s global protein families were employed [25]. The alignment of protein and gene sequences was conducted using muscle and BioPython [26,27].

### *In silico* analysis

Genome annotation was conducted using the DDBJ Fast Annotation and Submission Tool DFAST v1.2.0 [28]. Homology searches were performed using GENETYX-MAC v20.0.1 (Genetyx Corporation, Tokyo, Japan). The identification of secondary metabolite biosynthesis gene clusters was accomplished using online application antiSMASH, with a ‘relaxed’ strictness setting [29]. Genome islands were predicted using IslandViewer 4 [30].

### List of Abbreviation

A list of abbreviations of terms used in the manuscript is given in Table 1.

**Table 1.** List of abbreviations of terms used in the manuscript

Abbreviation	Term
ANI	average nucleotide identity
bp	base pair
dDDH	digital DNA–DNA hybridization
*E. coli*	*Escherichia coli*
MRSA	Methicillin-resistant *Staphylococcus aureus*
*P. aeruginosa*	*Pseudomonas aeruginosa*
*P. asplenii*	*Pseudomonas asplenii*
*P. batumici*	*Pseudomonas batumici*
*P. siliginis*	*Pseudomonas siliginis*
*S. aureus*	*Staphylococcus aureus*
spp.	Species
^T^	Type strain
VRSA	Vancomycin-resistant *Staphylococcus aureus*

## Results and discussion

### Isolation of Koz11

**Fig. 2.**
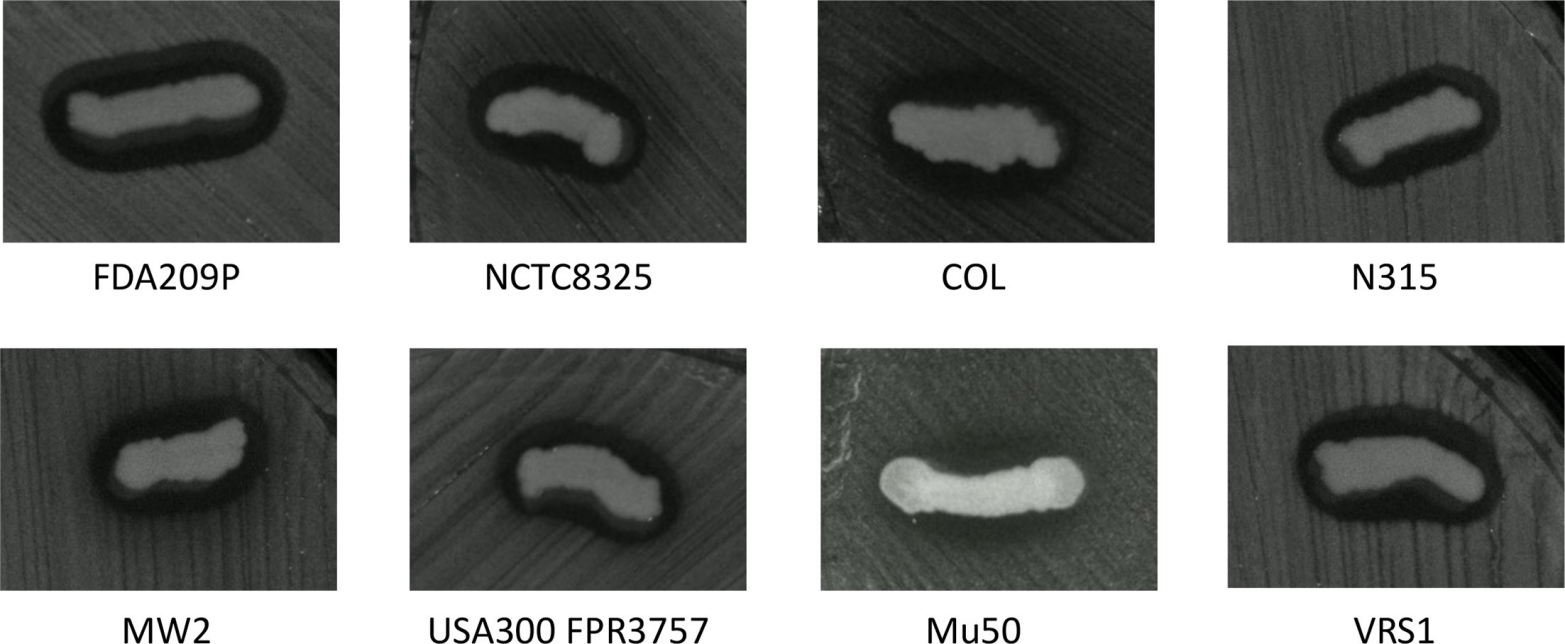
Inhibitory zones against *S. aureus* strains demonstrating antimicrobial activity of *P. batumici* Koz11.

Bacterial growth was observed in four of the six samples obtained from six different locations on Kozushima Island (Table 2). The numbers of colonies on these four plates were 1, 24, 68 and 13, respectively. From these four plate sites, 1, 4, 2 and 2 colonies were chosen for additional testing to determine their antimicrobial activity against a Gram-positive *S. aureus* strain FDA209P and a Gram-negative *E. coli* strain 1708. Following overnight incubation, one isolate, Koz11, exhibited an inhibitory zone against *S. aureus*, while none of the nine colonies produced an inhibitory zone against *E. coli*. Subsequently, the antimicrobial activity of Koz11 was tested against seven additional *S. aureus* strains, including the VRSA strain VRS1. An inhibitory zone was observed for each of the tested strains, with mean lengths ranging from 4.45 to 2.66 mm (Table 3, Fig. 2 and Fig. S2). Furthermore, the inhibition assay was performed using trypticase soy agar and Mueller–Hinton agar under the same cultural conditions. The formation of inhibition zones was smaller or non-existent with those media (Fig. S3). So far, we have not determined the key nutrients that promote or inhibit the antimicrobial activity of *P. batumici*. Therefore, further investigation into the experimental conditions is needed to determine the optimal conditions for Koz11 to produce antimicrobial compounds.

**Table 2.** Summary of *Pseudomonas* spp. isolated from soil samples obtained at six sites on Kozushima Island

Location numbers of the sites where soil samples were obtained	1	2	3	4	5	6
Numbers of colonies that:						
Grew on *Pseudomonas* spp. selective medium*	1	0	0	24	68	13
Had different morphological characteristics	1	0	0	4	2	2
Numbers of isolates that showed antimicrobial activity to:						
* S. aureus* FDA209P	0	–	–	1	0	0
* E. coli* 1708	0	–	–	0	0	0

1:, west-coast beach; 2:, southeast passage; 3:, southeast observatory; 4:, seventh climbing stage of Mt. Tenjo; 5:, north-coast beach; 6:, edge of pond on Mt. Tenjo summit;. CFC/CN agar.

* *Pseudomonas* CFC/CN agar.

**Table 3.** Antibiotic activity of *P. batumici* Koz11 against eight representative *S. aureus* strains

	Inhibition zone (mm)	Minimum inhibitory concentrations (mg/l)										
*Staphylococcus aureus* strains	**Koz11**	**OXA**	**CFX**	**FMOX**	**IPM**	**GM**	**ABK**	**EM**	**CLDM**	**LVFX**	**VCM**	**LZD**
FDA209P	4.45	≤0.12	≤4	≤0.5	≤0.25	0.5	1	0.25	0.12	0.5	≤0.5	1
NCTC8325	3.06	≤0.12	≤4	≤0.5	≤0.25	≤0.25	≤0.25	0.5	0.25	0.5	1	2
COL	2.66	＞4	>16	>16	>8	≤0.25	0.5	1	0.25	0.5	2	2
N315	4.30	＞4	>16	16	8	≤0.25	0.5	>4	>2	0.5	≤0.5	2
MW2	3.30	＞4	>16	8	2	≤0.25	0.5	0.5	0.25	0.5	1	2
USA300 FPR3757	3.55	＞4	>16	16	2	0.5	1	>4	>2	>4	1	2
Mu50	3.68	＞4	>16	>16	>8	>8	4	>4	>2	>4	8	1
VRS1	3.82	＞4	>16	>16	>8	>8	1	>4	>2	>4	>16	2

Numbers in the table are minimum inhibitory concentrations but for Koz11 which denote mean length (mm) from Koz11 growth to the edge of the inhibition zone as described in Fig. S2.

ABK, arbekacin; CFX, cefoxitin; CLDM, clindamycin; EM, erythromycin; FMOX, flomoxef; GM, gentamicin; IPM, imipenem; LVFX, levofloxacin; LZDlinezolidLZD, linezolidOXA, oxacillin; VCM, vancomycin

Koz11 was inoculated onto a nutrient agar plate using a sterilized toothpick where a pathogenic strain had been spread. An inhibition zone was observed after overnight incubation at room temperature, followed by 4 to 6 h at 37 °C to promote the growth of the pathogenic strain in the background.

### Species determination

Koz11 underwent species identification using 16S rRNA sequence data. The top three similarities with 100 % completeness among type strains from the 1460 bp of the 16S rRNA sequence were found with *P. batumici* UCM B-321^T^, *P. siliginis* SWRI31^T^ and * P. asplenii* ATCC 23835^T^, with similarity scores of 99.03%, 98.89% and 98.82%, respectively. While the 16S rRNA gene sequence is widely used for bacterial species determination, its taxonomic resolution is inadequate for *Pseudomonas* spp. [31], necessitating further investigation. Whole-genome sequencing of Koz11 was conducted, and the data were compared with closely related species. The generally accepted species boundaries are 95–96% for ANI and 70% for dDDH [32]. The analysis suggested that Koz11 belonged to *P. batumici,* with ANI and dDDH scores of 97.2 and 77.2%, respectively. The ANI and dDDH values for Koz11 and closely related species are presented in Table S1.

### Phylogenetic relationships of Koz11 strain and *Pseudomonas* type strains

**Fig. 3.**
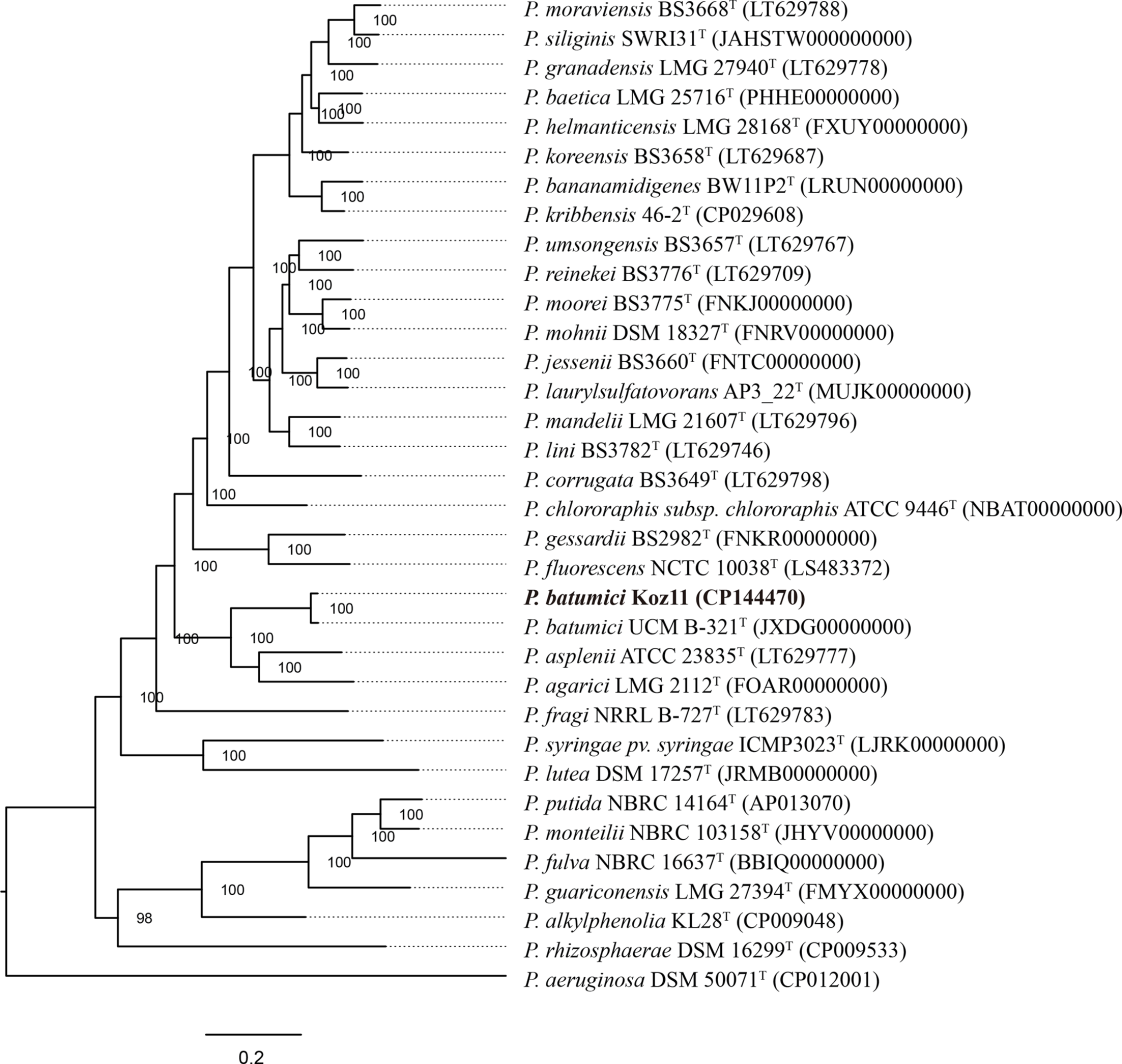
Phylogenetic analysis based on whole-genome sequences of strain Koz11 and 33 reference *Pseudomonas* strains. BV-BRC genome ID code, parameters and protein families (requested 1000) were used to create a tree. GenBank accession numbers are provided in parentheses. Koz11 is highlighted in bold.

The result of phylogenetic analysis based on the whole-genome sequence of Koz11 and 33 *Pseudomonas* type strains is shown in Fig. 3. Koz11 strain and *P. batumici* UCM B-321^T^ form a single clade, visually representing the genetic relatedness between Koz11 and UCM B-321^T^ among the 33 type strains.

### Chemotaxonomic and biochemical characterization

The major fatty acid profile and biochemical characteristics are essential for species characterization. Since the methods used to characterize *P. batumici* UCM B-321^T^ differ from the current standard methods [11], we conducted chemotaxonomic and biochemical analyses of Koz11 to contribute to the species’ taxonomy. The major fatty acids detected in Koz11 included C_10:0_ 3OH, C_12:0_ 2OH, C_12:1_ 3OH, C_12:0_ 3OH, C_16:00_, C_17:0_ cyclo, summed feature 2, summed feature 3 and summed feature 8 (Table S2). The fatty acid composition of *P. batumici* UCM B-321^T^ differs from that of Koz11 [11], notably in C_10:0_ 3OH, C_12:0_ 2OH, C_12:0_ 3OH and C_12:0_ 3OH, which are present at >5.0 % in Koz11 but are either missing or present at <1.0 % in UCM B-321^T^. These differences in fatty acid composition may be related to the diverse characteristics of the strains within the same species; however, they are more likely due to the differences in methodology and culture medium. In this study, we used trypticase soy agar plates for the preculture medium, which have been preferred in recent chemotaxonomic studies on *Pseudomonas* spp. [33,37], while peptone-meat-infusion agar was used to analyse the UCM B-321^T^ strain [11]. Biochemical analysis revealed that the strain could utilize d-mannitol, potassium gluconate and capric acid for its growth. Moreover, it thrived under conditions of pH ≥5.0 and the presence of 1 % NaCl. The results of the biochemical characteristics are presented in Table S3.

### *In silico* molecular analysis

*P. batumici* UCM B-321^T^ is known to produce an antimicrobial substance named batumin, a polyketide compound synthesized by a hybrid polyketide synthase/non-ribosomal peptide synthetase pathway [14]. We located the batumin gene cluster on the *P. batumici* UCM B-321^T^ genome (from 284 229 to 360 497 bp on JXDG01000003) to perform a homology search on the Koz11 genome. Although Koz11 exhibits antimicrobial activity, the batumin gene cluster was not detected in its genome. *In silico* genomic island identification suggested that all the genes comprising the batumin gene cluster, except Bat2 and Bat3, originated from a horizontally transferable genomic island, indicating that the batumin gene cluster may not be a common feature among * P. batumici* species. To identify alternative genes responsible for antimicrobial compounds in Koz11, we used the online secondary metabolism detection tool, antiSMASH, which identified 11 secondary metabolite regions in Koz11. These included putative streptophenazine, lassopeptides, ambactin, the plant pathogenic syringomycin and the anti-tumour compound oviedomycin gene. Streptophenazines with an N-formylglycine moiety have been reported to have antimicrobial activity against MRSA USA300 TCH1516 [38], and some lassopeptides are known to have antibiotic activity against pathogenic bacteria, including *S. aureus* [39]. Therefore, either one or both of these compounds may contribute to the antimicrobial activity of Koz11. Despite lacking the batumin gene cluster, Koz11 exhibits antimicrobial activity against *S. aureus* through a different mechanism. Co-isolation of * S. aureus* and *P. aeruginosa* from the same infection site is not uncommon [40,42]. *S. aureus* and *P. batumici*, which belong to the same genus as *P. aeruginosa*, may also co-exist and compete in the natural environment. The list of Bat genes of *P. batumici* UCM B-321^T^ identified by the genomic island finder is provided in Table S4, and the list of secondary metabolite regions of Koz11 and *P. batumici* UCM B-321^T^ is presented in Table S5.

### Geological and ecological significance of *P. batumici*

Several geographical similarities were observed between the two locations of *P. batumici* isolation. Mt. Tenjo, where Koz11 was sampled, is not directly on the coastline, but the small island of Kozushima (spanning only 18.24 km²) is influenced by coastal climates throughout its entirety. The climates of Kozushima and the Black Sea coast share common characteristics; both areas fall under the ‘Humid Subtropical’ classification according to the Köppen Climate Classification [43,44]. Additionally, both Kozushima and the South Caucasus are geochemically rich in obsidian [45,46]. Obsidian was a highly valuable resource for ancient societies [47], leading to numerous geological and archaeological studies in obsidian-rich regions [48,50]. These studies provide valuable information, including evidence of ancient human traffic through obsidian exchanges, which may contribute to further exploration of the *P. batumici* strains.

Kozushima is a volcanic island in the Izu archipelago, which is the sixth island from Japan’s main island, Honshu, with no direct access to foreign countries. Kozushima, believed to have formed in the Pleistocene era due to volcanic activity, has its oldest stratum estimated to be a relatively recent 80  000 years old [51]. This suggests that soil bacteria can be readily transferred through natural processes such as prevailing westerly winds, migratory birds or human activity, including migration within the Izu archipelago during the Palaeolithic era [52]. A soil ecosystem in one area may influence distant locations through dynamic circulation and exchange of soil organisms. While *P. batumici* demonstrates antagonism against antibiotic-resistant pathogenic strains, resistant strains that emerge in one location may become widespread. Further discovery of *P. batumici* strains is warranted to elucidate the distribution of species, contributing to a better understanding of ecological diversity, as well as exploring potential consequences of this strain within the context of soil health.

## supplementary material

10.1099/acmi.0.000799.v3 Uncited Fig. S1.

10.1099/acmi.0.000799.v3 Uncited Fig. S2.

10.1099/acmi.0.000799.v3 Uncited Fig. S3.

10.1099/acmi.0.000799.v3 Uncited Table S1.

## Abbreviations

ANI: average nucleotide identity
dDDH: digital DNA–DNA hybridization
MRSA: methicillin-resistant *Staphylococcus aureus*
*P. batumici*: *Pseudomonas batumici*
VRSA: vancomycin-resistant *Staphylococcus aureus*
