# Supplementary material for: Characteristics of soil origin Pseudomonas batumici Koz11 isolated from a remote island in Japan
Source: Access Microbiol. 2024 Aug 16;6(8):000799.v3. doi: 10.1099/acmi.0.000799.v3 (PMC11328868; doi:10.1099/acmi.0.000799.v3)
Supplement: Uncited Fig. S1. [file acmi-6-00799-s001.pdf]

Fig. S1a Experimental procedure for the isolation of Kozushima originated strains.

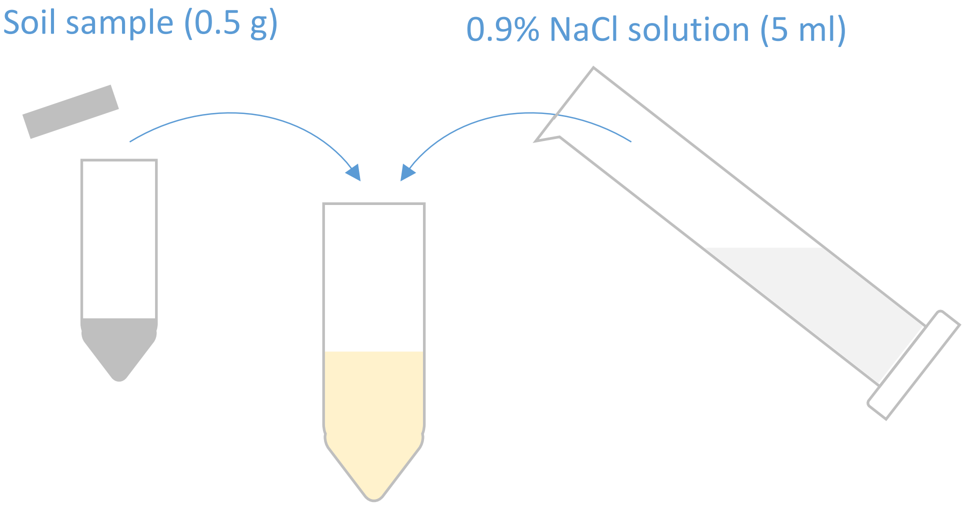

Approximately 0.5 g of each soil sample was suspended in 5 ml of 0.9% NaCl solution.

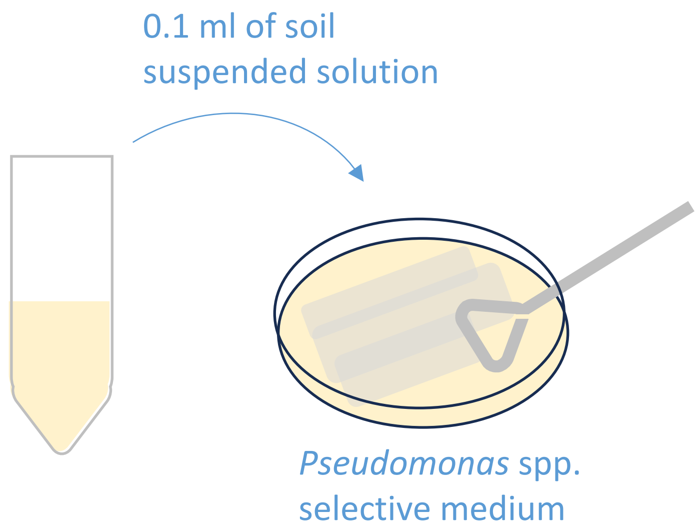

Next, 0.1 ml of the suspension from each sample was individually plated on *Pseudomonas* spp. selective medium.

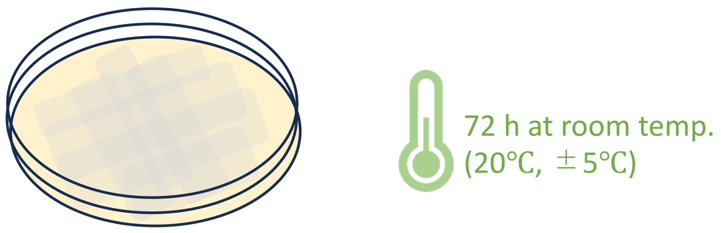

The plate was then incubated for 72 h at room temperature.

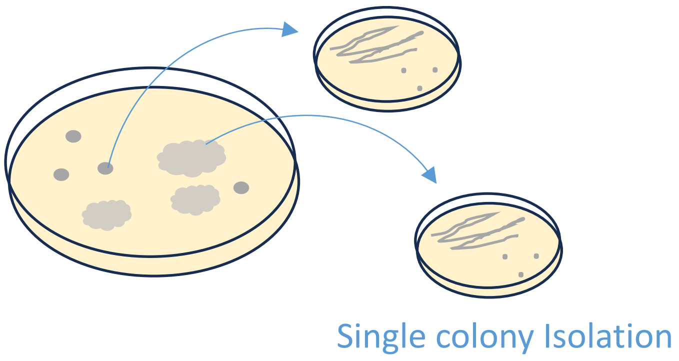

Colonies with different morphologies were selected and subjected to purification through the process of single colony isolation.

Fig. S1b The experimental procedure of the inhibition zone assay.

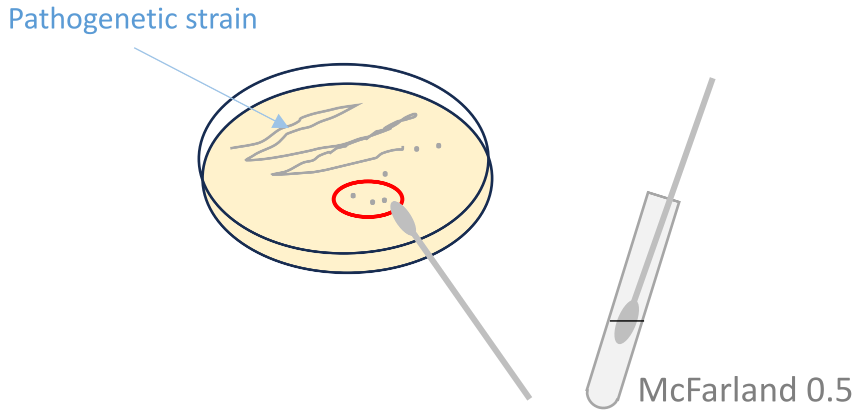

A few colonies of the pathogenetic strain on a nutrient agar were selected and suspended in saline, using a sterile swab.

Adjust the turbidity as McFarland standard of 0.5

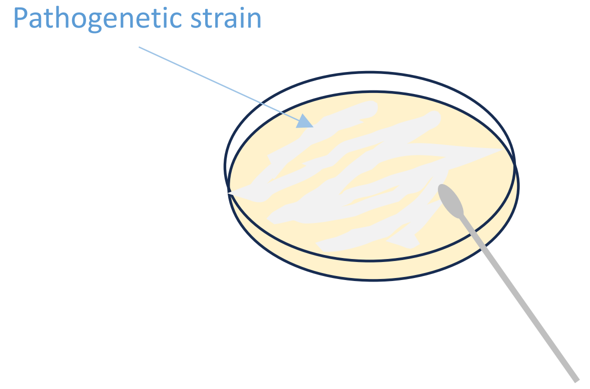

Spreading the pathogenetic strain on fresh nutrient agar medium with a swab.

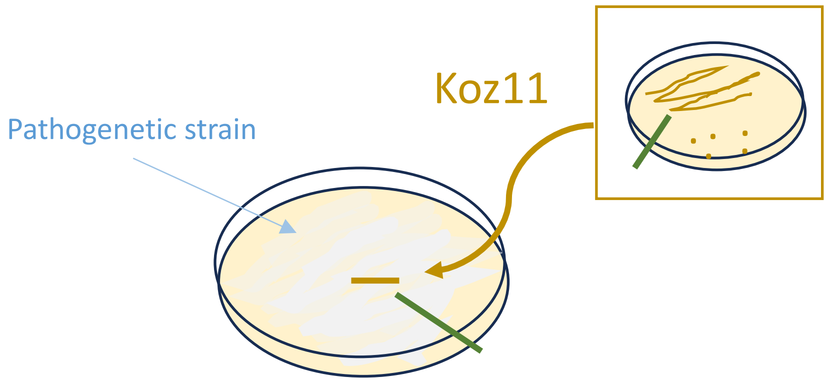

Koz11 colonies were transferred using a sterilized toothpick and inoculated onto a nutrient agar plate where a pathogenic strain had been spread.

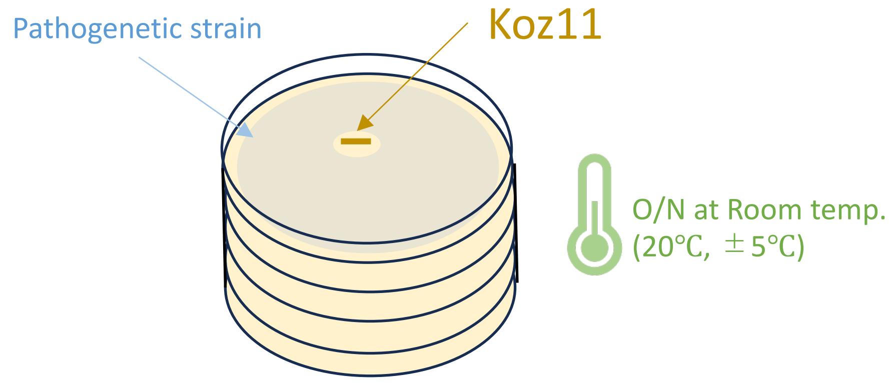

Incubate O/N at room temperature for 24h

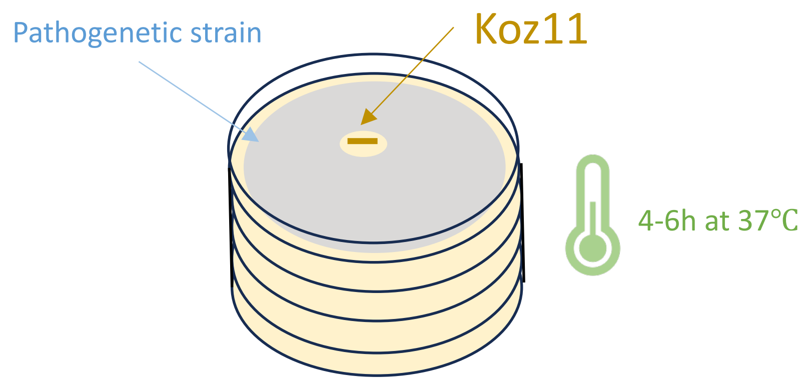

followed by an additional 4 to 6 hours at 37 C to promote the growth of the underlying pathogenic strain.

Fig. S1c The procedure to obtain the length of the inhibition zone

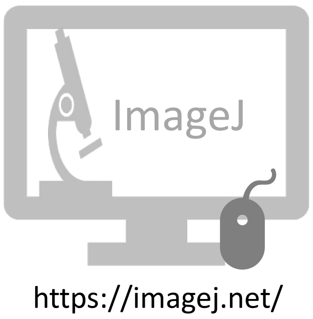

ImageJ, a free software provided by US National Institutes of Health, was used to measure the length of the inhibition zone.

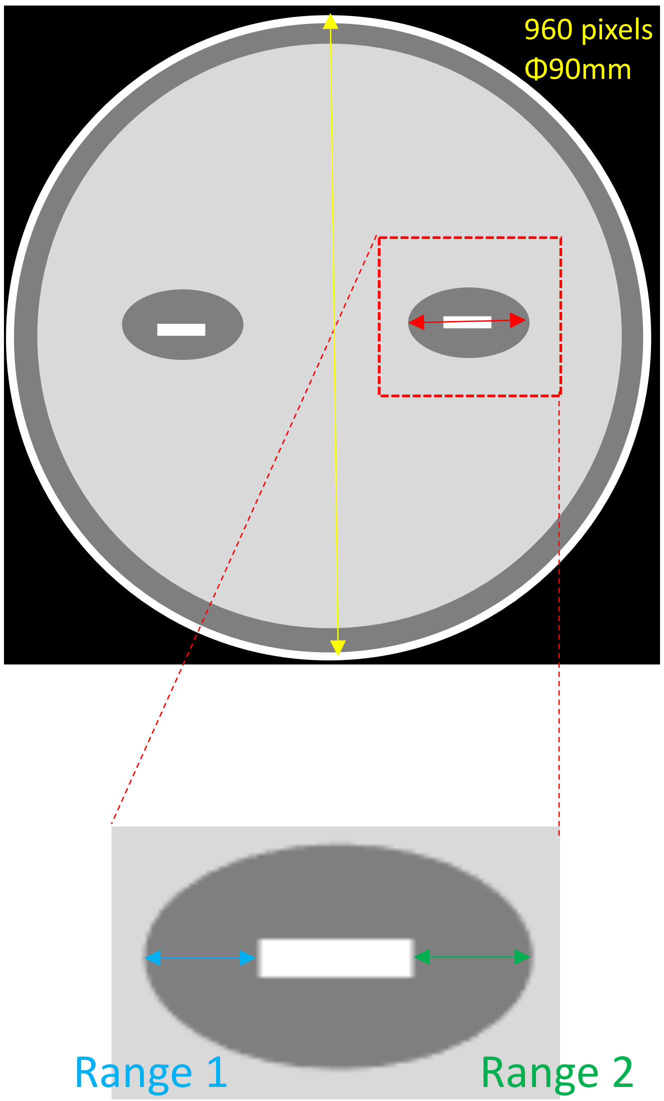

A pixel length from the horizontal edge of the inhibition zone was obtained using ImageJ's "plot file function".

The pixel distances from the horizontal edge of the inhibition zone and the edge of the Koz11 colony were measured.

The threshold value to determine the horizontal edge of Koz11 colony was identified at the position where the maximum value was obtained through differentiation.

The inhibition zone (Range 1 + Range 2) in mm was calculated by converting the pixel difference to millimeters, based on the diameter of the plate (960 pixels), which corresponds to an actual size of 90 mm.

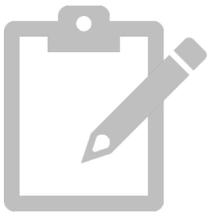

The average length is to be reported.

Example:
The extended length shown in red allow is 120 pixels, and the range 1 (blue allow) is from 0 to 20 pixels, and range 2 (green allow) is from 60 to 85 pixels. The pixel length is 45 pixels, which is equivalent to 4.23 mm.
