# Supplementary material for: Characteristics of soil origin Pseudomonas batumici Koz11 isolated from a remote island in Japan
Source: Access Microbiol. 2024 Aug 16;6(8):000799.v3. doi: 10.1099/acmi.0.000799.v3 (PMC11328868; doi:10.1099/acmi.0.000799.v3)

Fig. S2. Antibiotic activities of *Pseudomonas batumici* Koz11 against eight *Staphylococcus aureus* strains

FDA209P

	Zone 1	Zone 2	Zone 3	Zone 4	Zone 5	Zone 6	Zone 7	Zone 8
Total length (pixel)	88	104	116	110	79	81	87	80
Range 1 (pixel)	25	29	32	27	21	21	22	18
Range 2 (pixel)	25	26	32	27	19	18	20	18
Range 1 + 2 (pixel)	50	55	64	54	40	39	42	36
Range 1 + 2 (mm)	4.69	5.16	6.00	5.06	3.75	3.66	3.94	3.38
Average inhibition zone (mm)								4.45
SD								0.92

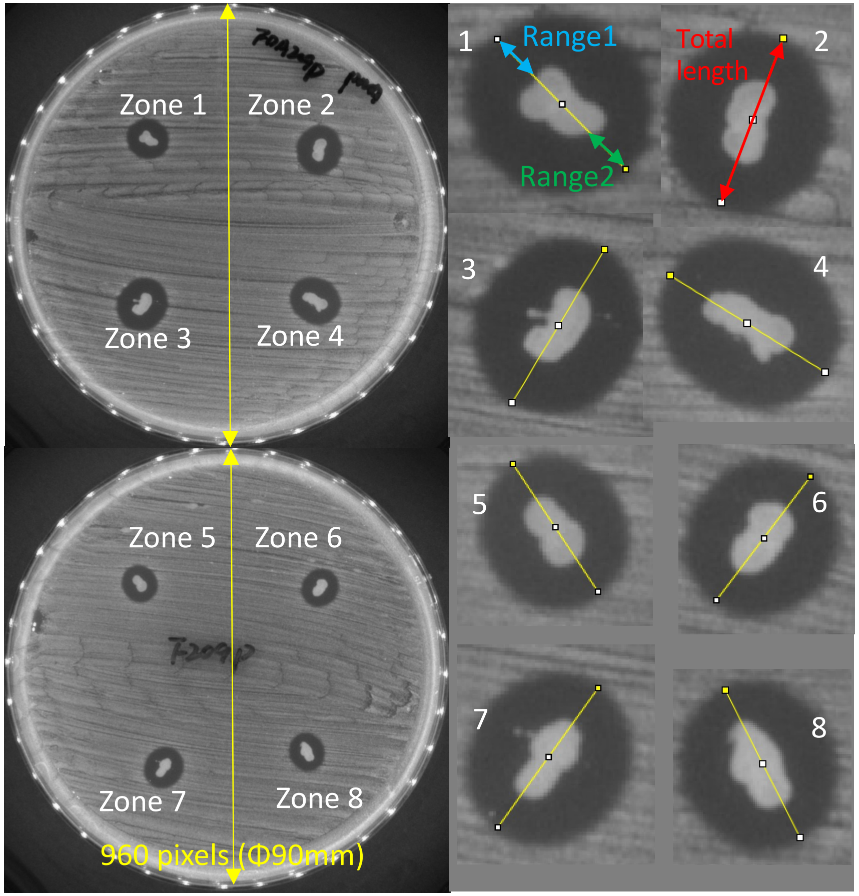

NCTC8325

	Zone 1	Zone 2	Zone 3	Zone 4	Zone 5	Zone 6	Zone 7	Zone 8
Total length (pixel)	79	87	81	73	78	78	65	70
Range 1 (pixel)	17	24	15	14	17	17	12	16
Range 2 (pixel)	15	21	21	15	15	15	13	14
Range 1 + 2 (pixel)	32	45	36	29	32	32	25	30
Range 1 + 2 (mm)	3.00	4.22	3.38	2.72	3.00	3.00	2.34	2.81
Average inhibition zone (mm)								3.06
SD								0.55

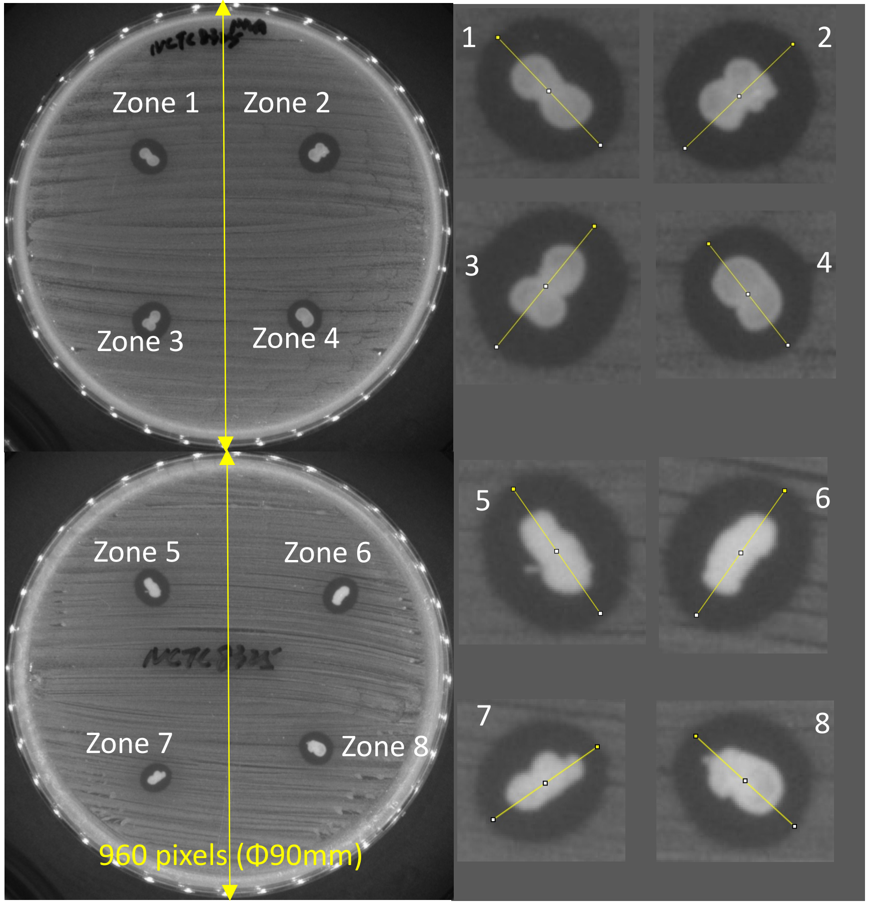

COL

	Zone 1	Zone 2	Zone 3	Zone 4	Zone 5	Zone 6	Zone 7	Zone 8
Total length (pixel)	69	67	76	78	67	65	64	71
Range 1 (pixel)	17	14	17	18	15	12	17	14
Range 2 (pixel)	12	13	15	17	12	10	12	12
Range 1 + 2 (pixel)	29	27	32	35	27	22	29	26
Range 1 + 2 (mm)	2.72	2.53	3.00	3.28	2.53	2.06	2.72	2.44
Average inhibition zone (mm)								2.66
SD								0.37

N315

	Zone 1	Zone 2	Zone 3	Zone 4	Zone 5	Zone 6	Zone 7	Zone 8
Total length (pixel)	95	107	93	92	91	84	75	80
Range 1 (pixel)	26	32	27	23	24	23	16	21
Range 2 (pixel)	23	28	26	22	21	21	15	19
Range 1 + 2 (pixel)	49	60	53	45	45	44	31	40
Range 1 + 2 (mm)	4.59	5.63	4.97	4.22	4.22	4.13	2.91	3.75
Average inhibition zone (mm)								4.30
SD								0.81

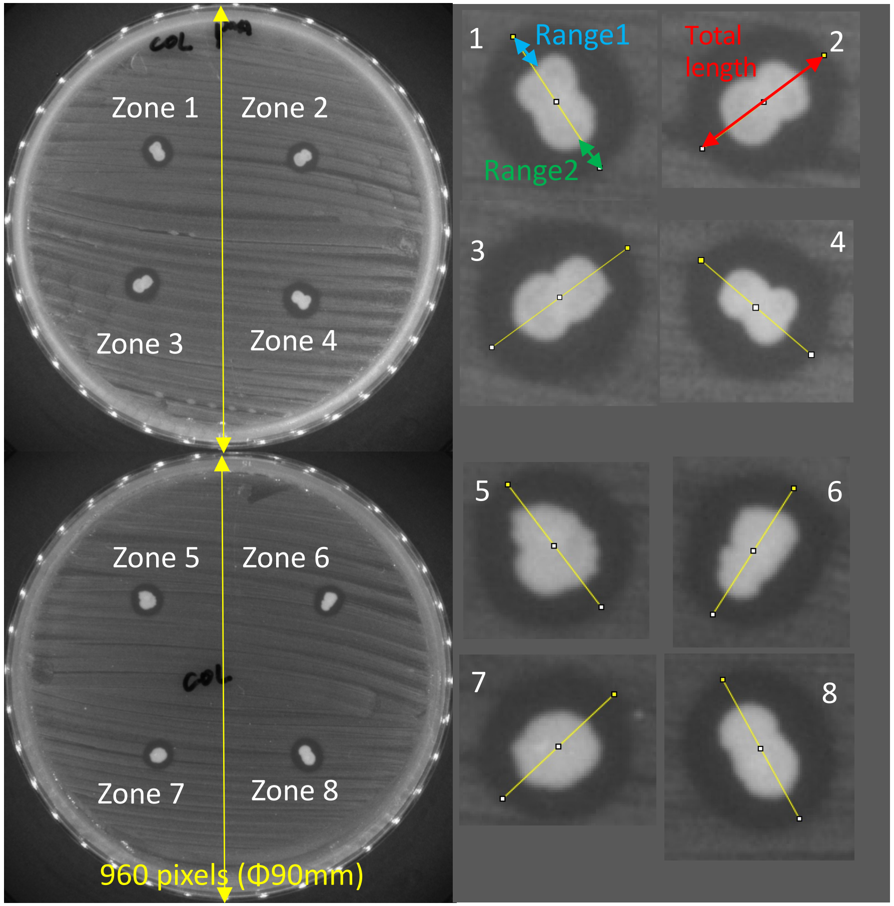

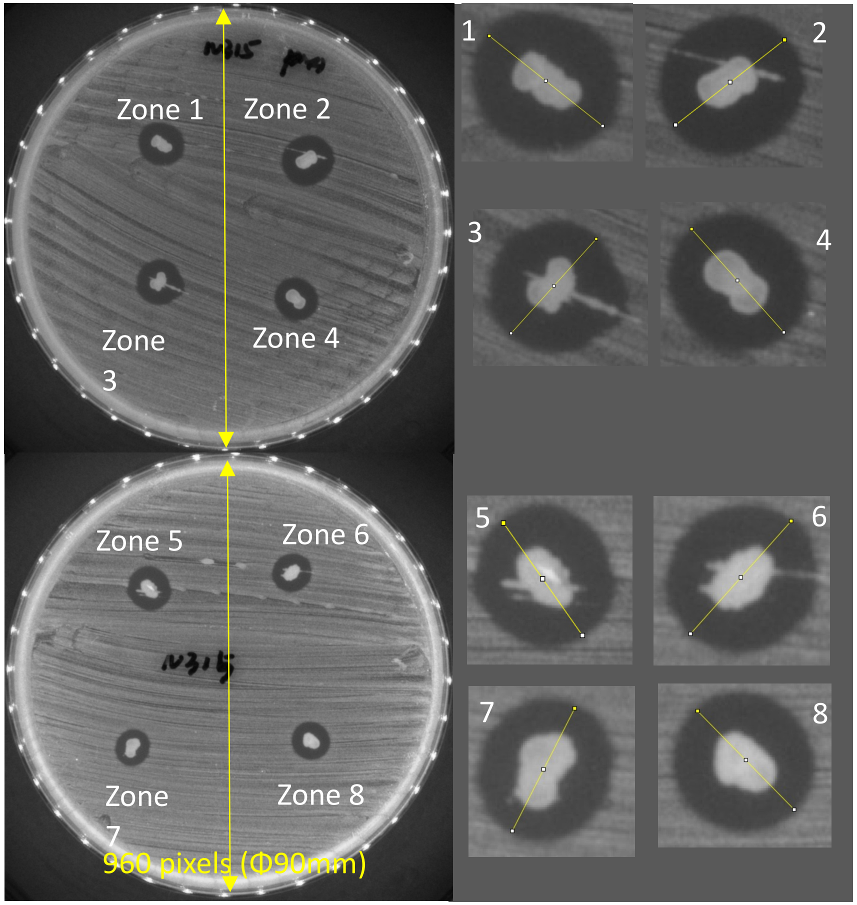

MW2

	Zone 1	Zone 2	Zone 3	Zone 4	Zone 5	Zone 6	Zone 7	Zone 8
Total length (pixel)	76	84	84	83	68	66	69	68
Range 1 (pixel)	21	22	22	20	17	16	17	15
Range 2 (pixel)	18	18	17	19	16	13	17	14
Range 1 + 2 (pixel)	39	40	39	39	33	29	34	29
Range 1 + 2 (mm)	3.66	3.75	3.66	3.66	3.09	2.72	3.19	2.72
Average inhibition zone (mm)								3.30
SD								0.43

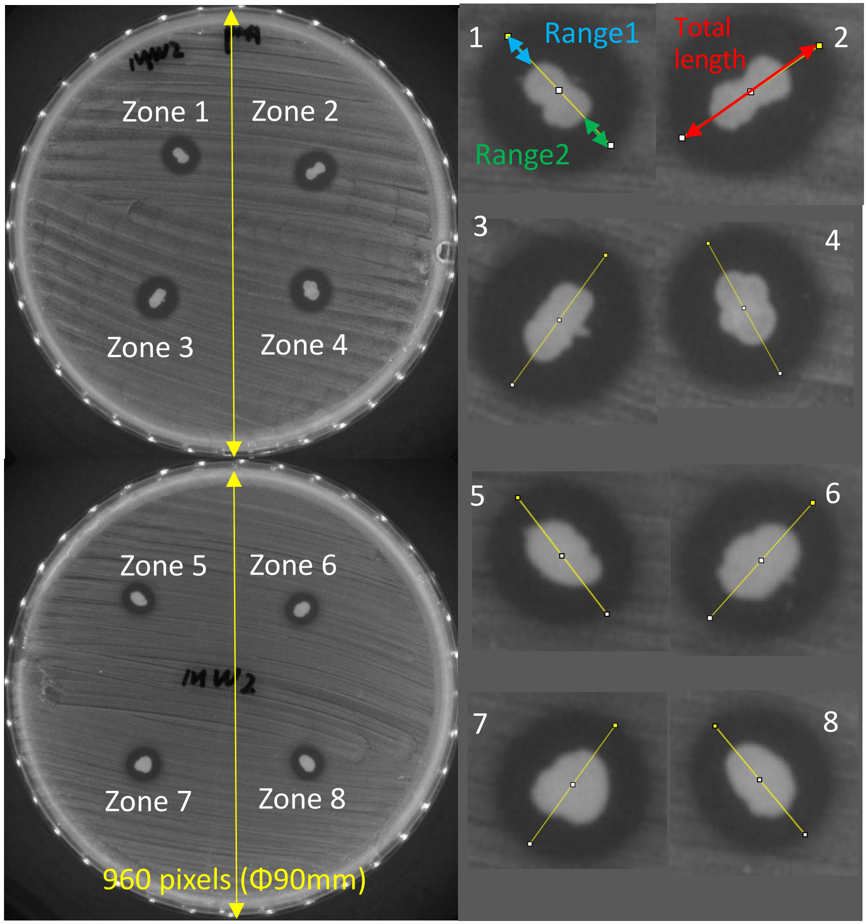

USA300-FPR3757

	Zone 1	Zone 2	Zone 3	Zone 4	Zone 5	Zone 6	Zone 7	Zone 8
Total length (pixel)	108	90	98	102	69	79	76	64
Range 1 (pixel)	27	21	20	24	18	15	16	14
Range 2 (pixel)	23	21	23	22	17	16	15	11
Range 1 + 2 (pixel)	50	42	43	46	35	31	31	25
Range 1 + 2 (mm)	4.69	3.94	4.03	4.31	3.28	2.91	2.91	2.34
Average inhibition zone (mm)								3.55
SD								0.81

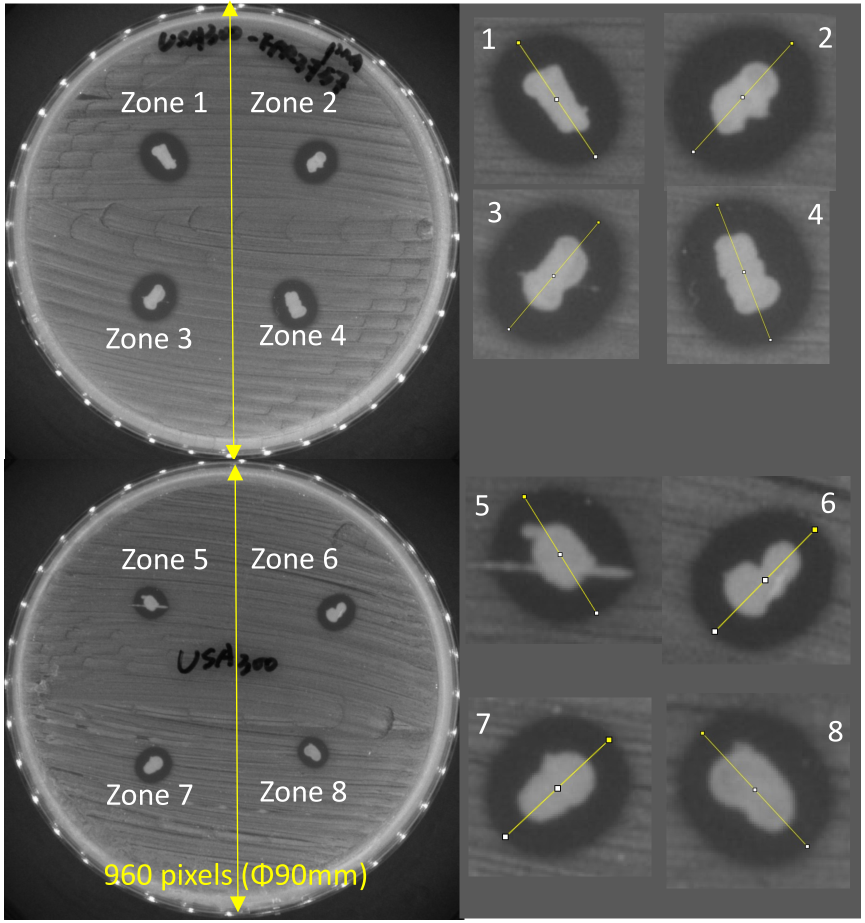

Mu50

	Zone 1	Zone 2	Zone 3	Zone 4	Zone 5	Zone 6	Zone 7	Zone 8
Total length (pixel)	99	86	104	100	78	88	77	74
Range 1 (pixel)	24	23	27	28	16	19	14	16
Range 2 (pixel)	22	18	20	26	15	15	15	16
Range 1 + 2 (pixel)	46	41	47	54	31	34	29	32
Range 1 + 2 (mm)	4.31	3.84	4.41	5.06	2.91	3.19	2.72	3.00
Average inhibition zone (mm)								3.68
SD								0.85

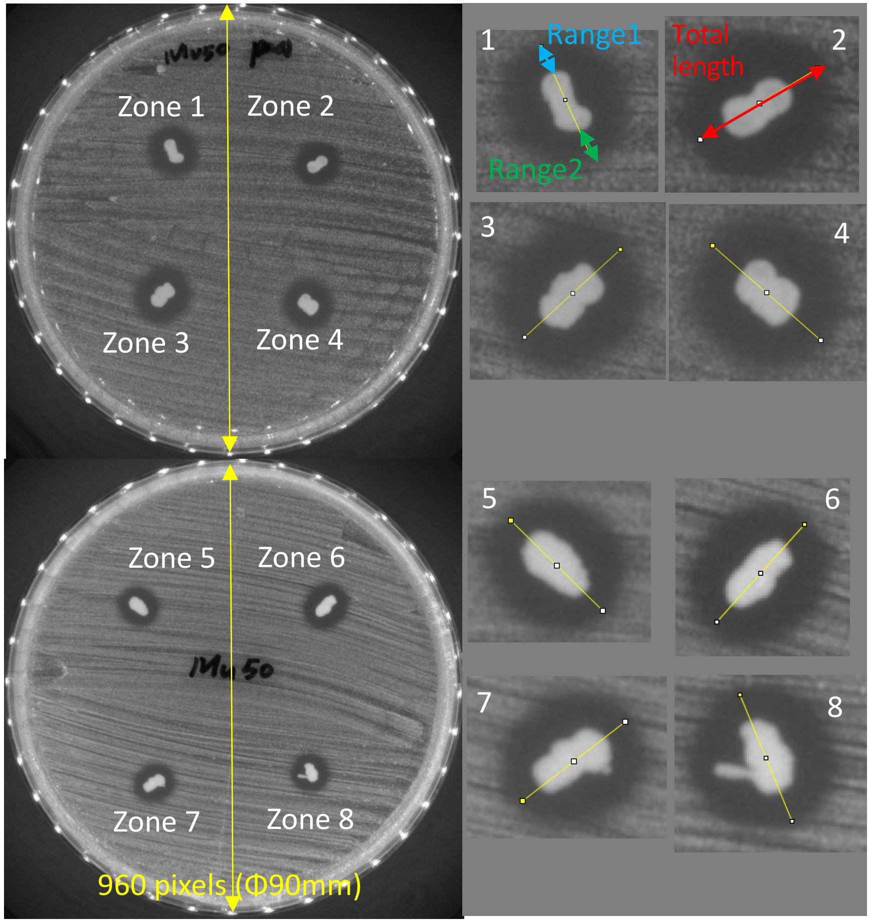

VRS1

	Zone 1	Zone 2	Zone 3	Zone 4	Zone 5	Zone 6	Zone 7	Zone 8
Total length (pixel)	103	112	102	101	65	67	74	72
Range 1 (pixel)	26	27	25	27	9	11	17	18
Range 2 (pixel)	28	25	27	26	15	14	16	15
Range 1 + 2 (pixel)	54	52	52	53	24	25	33	33
Range 1 + 2 (mm)	5.06	4.88	4.88	4.97	2.25	2.34	3.09	3.09
Average inhibition zone (mm)								3.82
SD								1.24

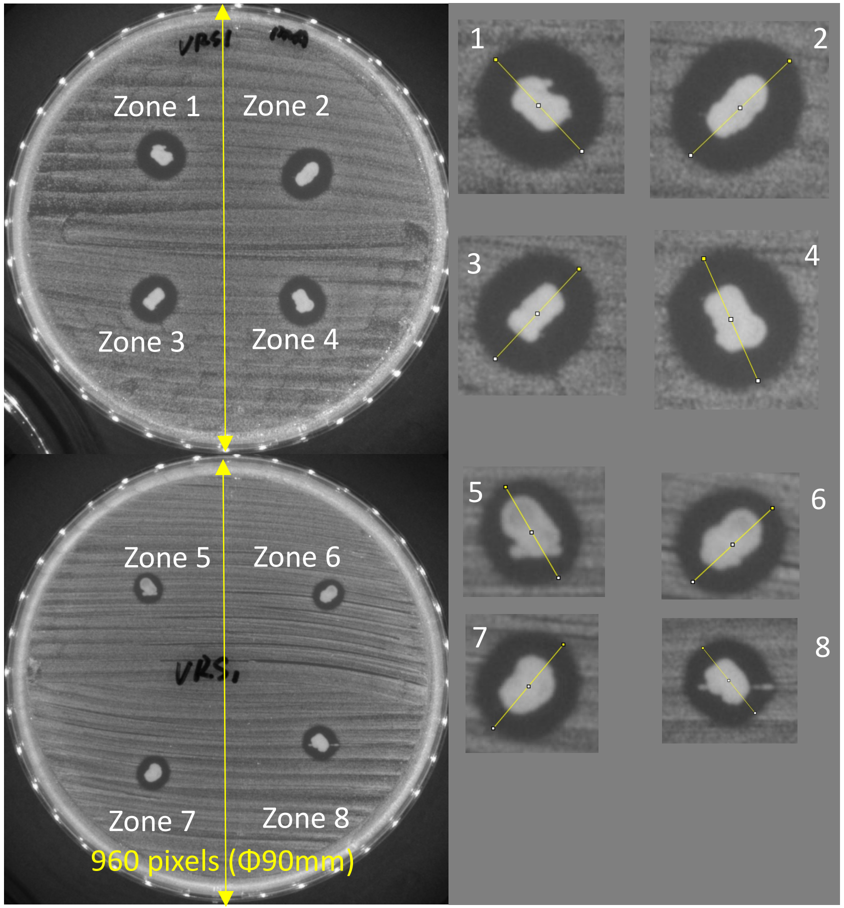

Supplement: Uncited Fig. S2. [file acmi-6-00799-s002.pdf]
