# Supplementary material for: Characteristics of soil origin Pseudomonas batumici Koz11 isolated from a remote island in Japan
Source: Access Microbiol. 2024 Aug 16;6(8):000799.v3. doi: 10.1099/acmi.0.000799.v3 (PMC11328868; doi:10.1099/acmi.0.000799.v3)

Figure S3 Comparison of the inhibition zone of Koz11 against eight *Staphylococcus aureus* strains by three different media

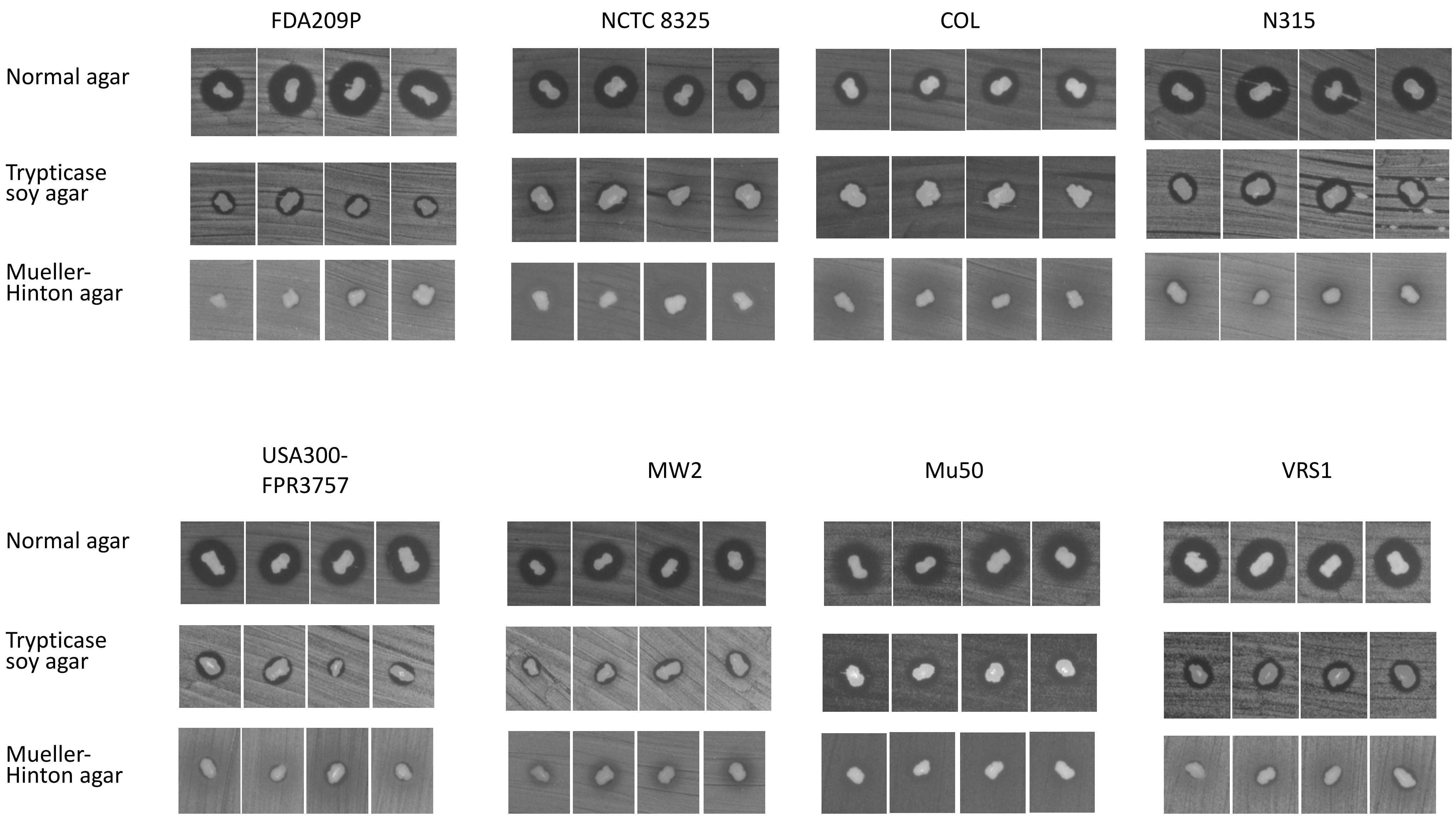

Supplement: Uncited Fig. S3. [file acmi-6-00799-s003.pdf]
