# Supplementary material for: Characteristics of soil origin Pseudomonas batumici Koz11 isolated from a remote island in Japan
Source: Access Microbiol. 2024 Aug 16;6(8):000799.v3. doi: 10.1099/acmi.0.000799.v3 (PMC11328868; doi:10.1099/acmi.0.000799.v3)
Supplement: Uncited Table S1. [file acmi-6-00799-s004.pdf]

Table S1. Average nucleotide identity (ANI) and digital DNA-DNA hybridization (dDDH) values of *Pseudomonas batumici (P. batumici)* Koz11 (CP144470) and related species

Strains	ANI (%)	dDDH (%)	GC content (%)	GenBank Accession number
P. batumici B-321^T	97.2	77.2	61.7	JXDG00000000
P. siliginis SWRI31^T	81.23	25.30	59.97	JAHSTW000000000
P. gingeri LMG 5327^T	86.0	31.4	62.6	POWE00000000
P. asplenni ATCC 23835^T	84.3	28.6	61.2	LT629777
P. fuscovaginae LMG 2158^T	84.3	28.6	61.4	LT629972
P. agarici LMG 2112^T	84.3	28.7	58.9	FOAR00000000
P. helmanticensis OHA11^T	81.0	25.0	59.2	CP027218

Table S2. Chemotaxonomic characterization of *P. batumici* Koz11

Fatty acids	***P. batumici* Koz11 (%)**
$C_{10:0}$ 3OH	13.41
$C_{12:0}$ 2OH	6.80
$C_{12:1}$ 3OH	6.67
$C_{12:0}$ 3OH	11.05
$C_{12:00}$	TR
$C_{16:00}$	23.81
$C_{17:0}$ cyclo	18.48
Summed Feature 2[1]	2.31
Summed Feature 3[2]	12.80
Summed Feature 8[3]	3.20

Fatty acids (> 1% of total fatty acids) are shown

[1]$C_{12:0}$ ALDE, unknown 10.928, $C_{16:1}$ ISO I, and $C_{14:0}$ 3OH

[2]$C_{16:1\omega7c}$ and $C_{16:1\omega6c}$

[3]$C_{18:1\omega7c}$ or $C_{18:1\omega6c}$

TR: Fatty acid amount<1%

Table S3. Phenotypic characteristics of *P. batumici* Koz11

	***P. batumici* Koz11**
Activity of enzymes (API 20 NE test)	
Potassium nitrate	-
L-arginine	-
Gelatin (bovine origin)	-
Growth on (API 20 NE test)	
D-mannose	-
D-mannitol	+
N-acetyl-glucosamine	-
Potassium gluconate	+
Capric acid	+
Phenylacetic acid	-
Carbon sources and growth condition (Biolog GN3)	
PH6	+
PH5	+
1% NaCl	+
4% NaCl	-
D-trehalose	+
Sucrose	+
α-D-glucose	w
D-mannose	w
D-fructose	w
D-galactose	-
D-fucose	-
Inosine	-
D-mannitol	w
D-arabitol	w
D-glucose6-PO4	w
D-fructose-6-PO4	w
D-aspartic acid	-
D-serine	-
Glycyl-L-proline	-
L-aspartic acid	+
Pectin	-
D-galacturonic acid	-
L-galactonic acid lactone	-
D-glucuronic acid	-
Glucuronamide	-
Mucic acid	-
Quinic acid	+
D-saccharic acid	-
p-hydroxy-phenylacetic acid	-
D-malic acid	+
Bromo-succinic acid	-
Tween 40	-
α-keto-butyric acid	-
Acetoacetic acid	-

+ positive, – negative, w weakly positive reaction

Table S4. List of genome islands of *P. batumici* UCM B-321^T (JXDG00000000)

Island start	Island end	Length	Gene name	Locus	Gene start	Gene end	Products
513919	520638	6719	KIH86004.1	UCMB321_0371	513919	520638	Bat1, batumin synthesis operon, polyketide synthase of type I
560324	591234	30910	KIH86007.1	UCMB321_0374	560324	560578	BatA, batumin synthesis operon, acyl carrier protein
560324	591234	30910	KIH86008.1	UCMB321_0375	560568	561785	BatB, batumin synthesis operon, 3-oxoacyl-(acyl-carrier-protein) synthase, KASII
560324	591234	30910	KIH86009.1	UCMB321_0376	561840	563078	BatC, batumin synthesis operon, 3-hydroxy-3methylglutaryl CoA synthase
560324	591234	30910	KIH86010.1	UCMB321_0377	563075	563842	BatD, batumin synthesis operon, methylglutaconyl-CoA hydratase
560324	591234	30910	KIH86011.1	UCMB321_0378	563839	564591	BatE, batumin synthesis operon, Enoyl-CoA hydratase
560324	591234	30910	KIH86012.1	UCMB321_0379	564717	566465	BatF, batumin synthesis operon, carbamoyl transferase
560324	591234	30910	KIH86013.1	UCMB321_0380	566584	567774	BatG, batumin synthesis operon, short-chain alcohol dehydrogenase family protein, trans-2-enoyl-CoA reductase
560324	591234	30910	KIH86014.1	UCMB321_0381	567894	568859	BatH, batumin synthesis operon, malonyl CoA-acyl carrier protein transacylase
560324	591234	30910	KIH86015.1	UCMB321_0382	568853	569650	BatI, batumin synthesis operon, 4'-phosphopantetheinyl transferase
560324	591234	30910	KIH86016.1	UCMB321_0383	569678	570544	BatJ, batumin synthesis operon, malonyl CoA-acyl carrier protein transacylase
560324	591234	30910	KIH86017.1	UCMB321_0384	570584	571960	BatK, batumin synthesis operon, malonyl CoA-acyl carrier protein transacylase or enoyl-(acyl-carrier-protein) reductase
560324	591234	30910	KIH86018.1	UCMB321_0385	572010	574175	BatL, batumin synthesis operon, hypothetical protein
560324	591234	30910	KIH86019.1	UCMB321_0386	574207	575142	BatM, batumin synthesis operon, short-chain dehydrogenase

Table S4. continued

Island start	Island end	Length	Gene name	Locus	Gene start	Gene end	Products
560324	591234	30910	KIH86020.1	UCMB321_0387	576045	577133	Fused batN and batO, batumin synthesis operon, Acyl-CoA dehydrogenase, short-chain specific
560324	591234	30910	KIH86021.1	UCMB321_0388	577130	579709	BatK, batumin synthesis operon, acetylornithine aminotransferase
560324	591234	30910	KIH86022.1	UCMB321_0389	579767	581218	BatQ, batumin synthesis operon, putative integral membrane protein
560324	591234	30910	KIH86023.1	UCMB321_0390	581229	582542	BatR, batumin synthesis operon, FAD dependent oxidoreductase
560324	591234	30910	KIH86024.1	UCMB321_0391	582583	584256	BatS, batumin synthesis operon, putative choline/carnitine o-acyltransferase
560324	591234	30910	KIH86025.1	UCMB321_0392	584273	585058	BatT, batumin synthesis operon, putative short-chain dehydrogenase
560324	591234	30910	KIH86026.1	UCMB321_0393	585078	586106	BatU, batumin synthesis operon, hypothetical protein
560324	591234	30910	KIH86027.1	UCMB321_0394	586123	587259	BatV, batumin synthesis operon, alkane-1 monooxygenase
560324	591234	30910	KIH86028.1	UCMB321_0395	587252	588073	BatW, batumin synthesis operon, putative fatty acid desaturase
560324	591234	30910	KIH86029.1	UCMB321_0396	588076	588333	BatX, batumin synthesis operon, putative acyl carrier protein
560324	591234	30910	KIH86030.1	UCMB321_0397	588433	590169	BatY, batumin synthesis operon, long-chain-fatty-acid--CoA ligase
560324	591234	30910	KIH86031.1	UCMB321_0398	590191	591234	BatZ, batumin synthesis operon, putative fatty acid desaturase

Table S5a. Secondary metabolite regions of *P. batumici* UCM B-321^{T} (JXDG00000000)

Region	Type	From	To	Most similar known cluster		Similarity (%)
Region 3.1	transAT-PKS,NRPS,PKS-like,T3PKS	270,960	360,854	Kalimantacin A (batumin)	NRP + Polyketide: Modular type I polyketide + Polyketide: Trans-AT type I polyketide	93
Region 4.1	RiPP-like	17,437	29,410			
Region 5.1	Polyketide (oviedomycin)	1	25,580	APE Vf	Other	40
Region 14.1	RiPP-like	20,171	31,061			
Region 21.1	Phosphonate	84,852	109,221			
Region 22.1	NRPS	1	8,869	MA026	NRP	6
Region 23.1	NRPS	1	15,432	Rhizomide A/Rhizomide B/ Rhizomide C	NRP	100
Region 26.1	NRPS-like	1	2,940	Rhizomide A/Rhizomide B/ Rhizomide C	NRP	100
Region 31.1	Hserlactone, NRPS	72,196	106,441	Corpeptin A/Corpeptin B	NRP	100
Region 34.1	NI-siderophore	44,757	56,607			
Region 39.1	NRPS	14,002	61,321	Brabantamide A	NRP: Lipopeptide + Saccharide: Hybrid/tailoring saccharide	60
Region 46.1	RiPP-like	69,437	81,055			
Region 50.1	NRPS-like	1	16,579	Nunapeptin/Nunamycin	NRP	50
Region 67.1	RiPP-like	77,854	88,699			
Region 68.1	NRPS-like	1	26,220	Fragin	NRP	37
Region 71.1	NRPS-like	1	6,267	Icosalide A/Icosalide B	NRP: Lipopeptide	100
Region 91.1	NRPS	1	3,968	Rhizomide A/Rhizomide B/ Rhizomide C	NRP	100
Region 97.1	Phenazine,hserlactone,arylpolyene	34,173	72,178	Pyocyanine	Other	100

Table S5b. Secondary metabolite regions of *P. batumici* Koz11 (CP144470)

Region	Type	From	To	Most similar known cluster		Similarity (%)
Region 1	NRPS,hserlactone	313,645	459,078	Syringomycin	NRP	100
Region 2	Lassopeptide	1,336,947	1,359,312			
Region 3	RiPP-like	1,808,292	1,818,258			
Region 4	RiPP-like	2,444,238	2,452,951			
Region 5	Polyketide (oviedomycin)	3,331,271	3,374,873	APE Vf	Other	40
Region 6	Hserlactone,phenazine	3,504,257	3,526,666	Streptophenazine B/Streptophenazine C/ Streptophenazine F/Streptophenazine G/ Streptophenazine H	NRP + Polyketide	21
Region 7	NRPS-like	3,652,941	3,696,384	Ambactin	NRP	25
Region 8	RiPP-like	3,838,323	3,849,168			
Region 9	Hserlactone	6,310,339	6,330,962			
Region 10	PKS-like	6,366,739	6,408,031	Orfamide A/Orfamide C	NRP: Cyclic Depsipeptide	17
Region 11	Siderophore	6,446,091	6,457,941			
